# Comparative chloroplast genomics and insights into the molecular evolution of *Tanaecium* (Bignonieae, Bignoniaceae)

**DOI:** 10.1038/s41598-023-39403-z

**Published:** 2023-08-01

**Authors:** Annelise Frazão, Verônica A. Thode, Lúcia G. Lohmann

**Affiliations:** 1 Departamento de Botânica, Instituto de Biociências, Universidade de São Paulo, São Paulo, SP Brazil; 2 Departamento de Biodiversidade e Bioestatística, Instituto de Biociências, Universidade Estadual Paulista, Botucatu, SP Brazil; 3 Programa de Pós-Graduação em Botânica, Departamento de Botânica, Instituto de Biociências, Universidade Federal do Rio Grande do Sul, Porto Alegre, RS Brazil; 4 Department of Integrative Biology, University and Jepson Herbaria, University of California, Berkeley, Berkeley, CA USA

**Keywords:** Molecular evolution, Plant sciences

## Abstract

Species of *Tanaecium* (Bignonieae, Bignoniaceae) are lianas distributed in the Neotropics and centered in the Amazon. Members of the genus exhibit exceptionally diverse flower morphology and pollination systems. Here, we sequenced, assembled, and annotated 12 complete and four partial chloroplast genomes representing 15 *Tanaecium* species and more than 70% of the known diversity in the genus. Gene content and order were similar in all species of *Tanaecium* studied, with genome sizes ranging between 158,470 and 160,935 bp. *Tanaecium* chloroplast genomes have 137 genes, including 80–81 protein-coding genes, 37 tRNA genes, and four rRNA genes. No rearrangements were found in *Tanaecium* plastomes, but two different patterns of boundaries between regions were recovered. *Tanaecium* plastomes show nucleotide variability, although only *rpo*A was hypervariable. Multiple SSRs and repeat regions were detected, and eight genes were found to have signatures of positive selection. Phylogeny reconstruction using 15 *Tanaecium* plastomes resulted in a strongly supported topology, elucidating several relationships not recovered previously and bringing new insights into the evolution of the genus.

## Introduction

The chloroplast is a circular organelle with a prokaryotic origin in plant cells. This organelle is responsible for photosynthesis and critical for the biosynthesis of starch, fatty acids, pigments, and amino acids^1,2^. Chloroplast genomes, also known as plastomes, have a predominantly conserved quadripartite structure that consists of a Large Single-Copy (LSC), two Inverted Repeats (IR), and a Small Single-Copy (SCC) region^3,4^. Despite the constancy in the overall structure, different patterns, rearrangements, structure organization, size, gene content, and order have been documented during the last decade^5–7^.

The structural variation observed in plastomes is due to intergenic region length and gene number, among others^9,10^. While closely related lineages tend to show lower variation, many cases of closely related species with high variation in plastome sizes have been observed^9,10^. This is probably associated with parasitism, IR loss, expansions, or contractions^7–9^. The increasing number of studies focusing on various plant clades adds publicly available data, allowing plastome comparisons among different angiosperm clades.

During the past three decades, chloroplast data has been extensively used to reconstruct plant phylogenies at different taxonomic levels^11–17^. The broad use of chloroplast data in molecular phylogenetic studies is due to its haploid nature, predominant uniparental inheritance, relatively stable gene structure, and high copy number per cell, which facilitates sequencing. While chloroplast sequencing initially targeted a few genes through Sanger approaches, the development of High-Throughput Sequencing (HTS) technologies allowed for whole plastome sequencing^18^.

The fast increase of HTS applications in the last couple of decades revolutionized the use of genomic data to understand the evolutionary history of green plants. In the tribe Bignonieae specifically, phylogenies reconstructed using plastome data have led to strongly supported and well-resolved topologies^16,19,20^. These phylogenies have improved our understanding of phylogenetic relationships at deep taxonomic levels (i.e., phylogeny backbone) and more recent divergences at the infra-generic level^16,20^.

*Tanaecium* Sw. emend. L.G. Lohmann (Bignonieae, Bignoniaceae) is a genus of Neotropical lianas that includes 21 species distributed from Mexico and the Antilles to Argentina, and centered in the Amazon^21^. The genus exhibits exceptionally diverse flower morphology and pollination systems^21^, seeds that can be winged or wingless and corky, and bromeliad-like prophylls of the axillary buds, a putative vegetative synapomorphy^21^. The genus was first sampled in a molecular phylogeny reconstructed using the chloroplast gene *ndh*F and the nuclear *pep*C^12^. Subsequent molecular phylogenetic studies with this group used the same molecular markers^22–24^. While representatives of this genus have been sampled in multiple studies, sampling remains limited, even lacking sampling of the type species of the genus. Moreover, the *Tanaecium* plastome structure has never been explored. Even though a study reported data on the plastome of *T. tetragonolobum* (Jacq.) L.G.Lohmann^25^, this plastome turned out to be *Callichlamys latifolia* (Rich.) K.Schum.^26^.

In Bignoniaceae, plastomes range from 150,154 bp in *Incarvillea compacta* Maxim.^27^ to 183,052 bp in *Bignonia magnifica* W.Bull*,* the latter representing the largest Lamiid plastome known to date^28^. Bignoniaceae plastomes also show structural rearrangements, such as the loss of the *ycf4* gene reported for *Adenocalymma*^20^, and variation in gene number, ranging from 110 to 157 genes^16,19,20,25,27–29^.

This study aims to increase our knowledge of Bignoniaceae plastome structure and evolution and bring new insights into the evolutionary history of *Tanaecium* by reporting on the plastome structure of the genus for the first time. To achieve this goal, we (1) sequenced and assembled complete or nearly complete plastomes of 15 species of *Tanaecium*, representing more than 70% of the known diversity in the genus 21; (2) characterized the overall plastome structure; (3) performed comparative genomic analyses; (4) identified putative repeats; (5) investigated patterns of selection in the chloroplast genes; and (6) reconstructed a phylogeny for *Tanaecium* using the newly assembled plastomes.

## Results

### Plastome assembly and characteristics

**Figure 1:**
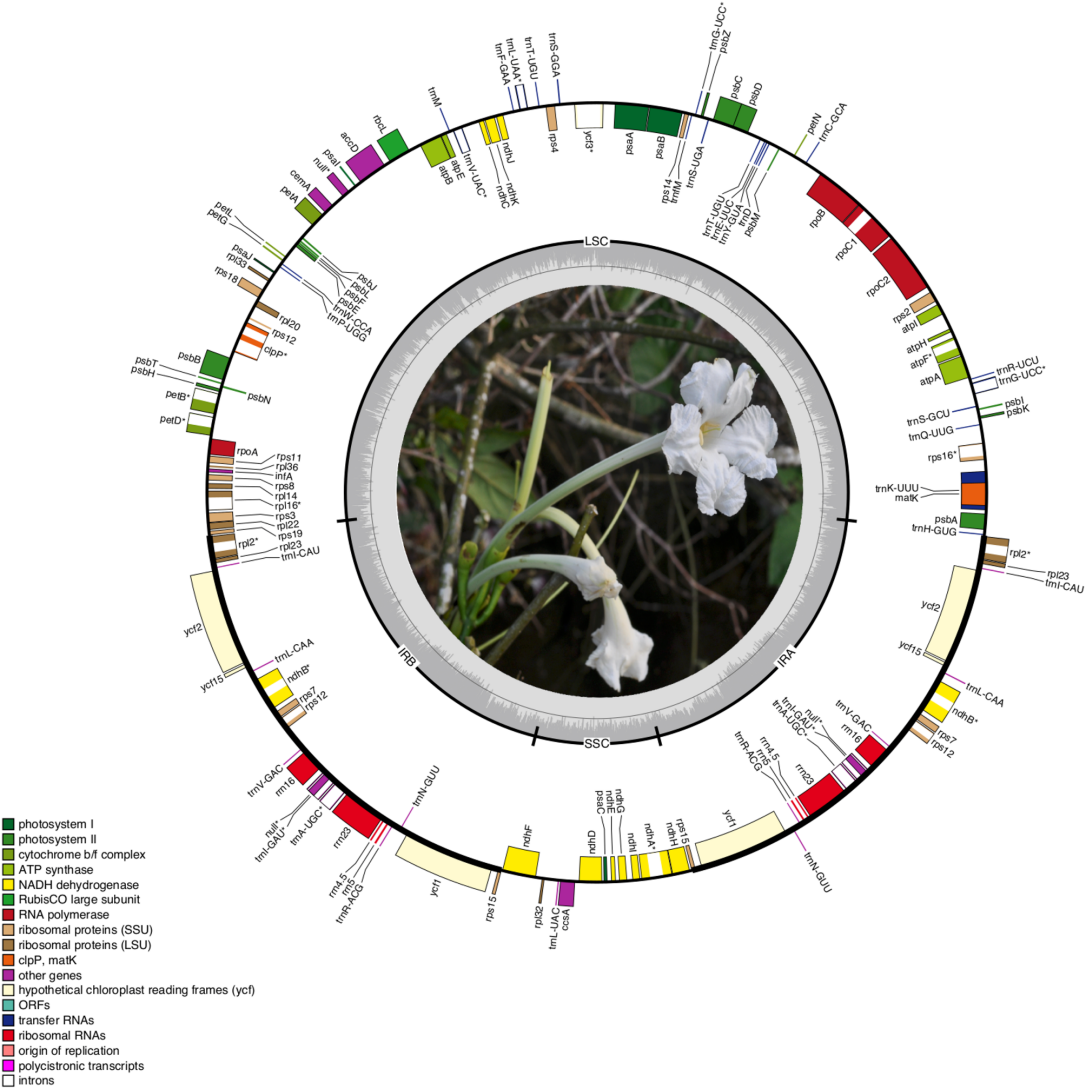
Representation of the plastome of *Tanaecium jaroba*. Genes drawn below the line are transcribed in a forward direction, while those drawn above the line are transcribed in a reverse direction. Asterisks (*) represent intron-containing genes.

**Figure 2:**
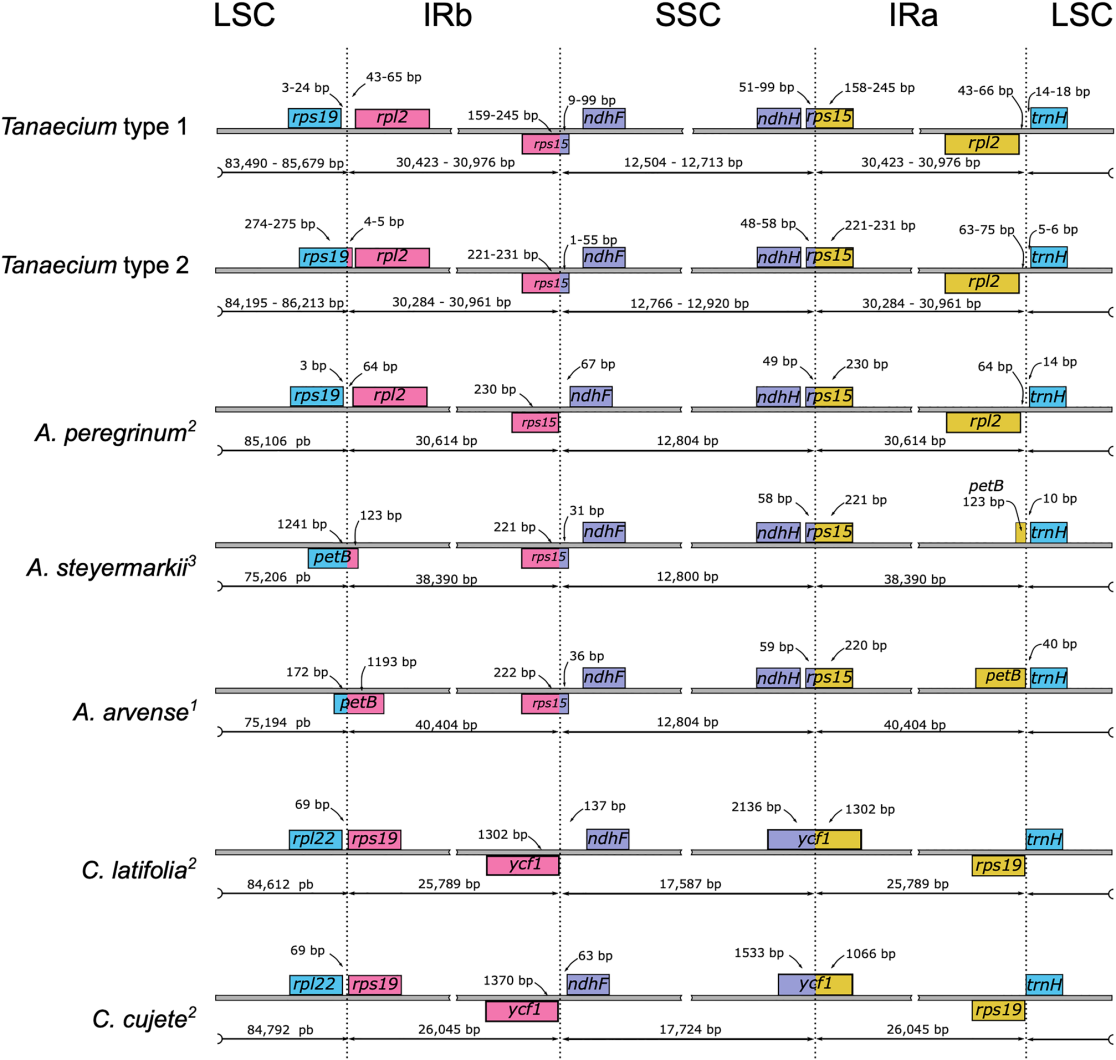
Comparison of the Large Single Copy (LSC), Inverted Repeat a (IRa), Small Single Copy (SSC), and Inverted Repeat b (IRb) boundaries within *Tanaecium* and among five other Bignoniaceae plastomes. The psi (*ψ*) indicates pseudogenes within the plastomes sampled. Genes shown below are transcribed reversely and those shown above the lines are transcribed forward. Minimum and maximum sizes for the regions and genes in the plastome boundaries are indicated in base pairs (bp). Numbers in superscript represent the literature from where the plastome boundary information were consulted. *Tanaecium* type 1 = *T. bilabiatum*, *T. crucigerum*, *T. cyrtanthum*, *T. decorticans*, *T. jaroba*, *T. parviflorum*, *T. pyramidatum*, *T. tetragonolobum*, *T. tetramerum*, and *T. truncatum*; *Tanaecium* type 2 = *T. dichotomum 1*, *T. dichotomum 2*, *T. duckei*, *T. revillae*, *T. selloi,* and *T. xanthophyllum*.

The paired-end raw reads of the 16 *Tanaecium* plastomes sequenced (Table 1) varied between 3,858,109 and 14,350,498 bp for *T. parviflorum* and *T. tetragonolobum*, respectively (Table 2). Of these, 12 plastomes were complete and four were partial. Mapped reads varied from 101,125 to 660,086 bp for *T. duckei* and *T. revillae*, respectively (Table 2). The average read depth varied between 85 × for *T. tetragonolobum* and 679 × for *T. dichotomum* 2 (Table 2). All plastomes showed the typical quadripartite structure of angiosperms (Fig. 1), with a pair of IR regions that range from 30,284 bp (*T. duckei*) to 31,089 bp (*T. bilabiatum*), intercalated by one LSC region that ranges from 83,490 bp (*T. crucigerum*; nearly complete, but without missing data in the LSC) to 86,213 bp (*T. xanthophyllum*), and one SSC region that ranges from 12,504 bp (*T. tetragonolobum*) to 12,920 bp (*T. dichotomum* 1) (Table 2). The *Tanaecium* plastomes have an average length of 159,359 bp, with *Tanaecium xanthophyllum* representing the largest plastome assembled here, with a total length of 160,935 bp (Table 2). The large size of the *T. xanthophyllum* plastome is due to an expansion in the LSC region (Table 2). The interquartile range (IQR) and median size ratio for *Tanaecium* was 0.5%; in turn, the IQR reported for *Adenocalymma* was 0.7%, for *Anemopaegma* was 0.4%, and for *Amphilophium* was 4% as expected based on an earlier study^9^ (Supplementary Table S7). The average GC content is 38% for all *Tanaecium* species studied (Table 2). All plastomes encode 137 genes, including 80–81 unique coding genes (CDS) (9 duplicated), 37 tRNA genes, and four rRNA genes (Tables 2 and 3). The Mauve analysis retrieved a single synteny block, indicating no rearrangements in *Tanaecium* plastomes (Supplementary Fig. S1). The boundaries between the chloroplast main regions are similar within *Tanaecium*, except for the LSC/IRb border, which can be located between the genes *rps*19 and *rpl2* or within the *rps*19 gene (Fig. 2).

**Table 1:** Taxa, voucher, reference, and GenBank accession numbers of the samples analyzed in this study.

Taxa	Voucher (collection)	GenBank accession number and reference
*Adenocalymma peregrinum*	Fonseca 444 (SPF)	MG008314^20^
*Amphilophium steyermarkii*	Steyermark 106874 (MO)	MK163626^16^
*Anemopaegma arvense*	Firetti 241 (SPF)	MF460829^19^
*Callichlamys latifolia*	Lohmann 619 (MO, SPF)	KR534325^25^
*Crescentia cujete*	Not informed	KT182634^29^
*Tanaecium bilabiatum*	Lohmann 850 (SPF)	OP218850
*Tanaecium crucigerum*	Lohmann 355 (SPF, MO)	OP218851
*Tanaecium cyrtathum*	Frazão 173 (SPF)	OP218852
*Tanaecium decorticans*	Frazão 188 (SPF)	OP218853
*Tanaecium dichotomum 1*	Frazão 375 (SPF)	OP218854
*Tanaecium dichotomum 2*	Carvalho 14 (SPF)	OP218855
*Tanaecium duckei*	Frazão 309 (SPF)	OP218856
*Tanaecium jaroba*	Frazão 288 (SPF)	OP218857
*Tanaecium parviflorum*	Fonseca 280 (SPF)	OP218858
*Tanaecium pyramidatum*	Fonseca 321 (SPF)	OL782596
*Tanaecium revillae*	Kataoka 321 (SPF)	OP218859
*Tanaecium selloi*	Frazão 235 (SPF)	OP218860
*Tanaecium tetragonolobum*	Frazão 419 (SPF)	OP218861
*Tanaecium tetramerum*	Pace 31 (SPF)	OP169019
*Tanaecium truncatum*	Frazão 340 (SPF)	OP169020
*Tanaecium xanthophyllum*	Frazão 333 (SPF)	OP169021

**Table 2:** Summary of sequenced plastomes of *Tanaecium*. In bold, nearly complete plastomes. *LSC* Large Single Copy, *IR* Inverted repeat, *SSC* Small Single Copy. *Approximate size. #: *acc*D partial; $: *clp*P partial; £: *rps*15 and *ndh*F partials, with IRb and SSC sizes undetectable.

Species	Voucher	No. of raw reads	No. of mapped reads	Average reads depth (x)	Plastome length (bp)	LSC length (bp)	IR length (bp)	SSC length (bp)	GC content (%)	Total CDS	Unique CDS	tRNA	rRNA	Genes
*T. bilabiatum*	Lohmann 850	8,860,486	447,380	424	159.587	84.647	31.089	12.762	38.1	90	81	37	8	137
***T. crucigerum***^**#,$**^	Lohmann 355	13,758,337	293,168	278	157.807*	83.490*	30.861	12.595	37.9	90	81	37	8	137
*T. cyrtanthum*	Frazão 173	11,648,305	467,930	482	159.444	85.068	30.899	12.578	38	90	81	37	8	137
*T. decorticans*	Frazão 188	12,644,022	306,343	297	159.241	85.259	30.648	12.686	38.1	90	81	37	8	137
*T. dichotomum 1*	Frazão 375	12,082,406	464,896	489	158.470	84.808	30.371	12.920	38	90	81	37	8	137
*T. dichotomum 2*	Carvalho 14	11,994,696	470,798	679	158.718	85.054	30.412	12.840	38	90	81	37	8	137
*T. duckei*	Frazão 309	11,812,767	101,125	97	158.751	85.414	30.284	12.769	38	90	81	37	8	137
*T. jaroba*	Frazão 288	11,096,666	422,891	439	160.061	85.679	30.894	12.594	37.9	90	81	37	8	137
***T. parviflorum***^**£**^	Fonseca 280	3,858,109	150,583	149	159.004*	85.272	–	–	38	90	81	37	8	137
*T. pyramidatum*	Fonseca 321	12,089,468	319,715	160	160.112	85.651	30.976	12.509	38.1	90	81	37	8	137
***T. revillae***^**#**^	Kataoka 321	10,642,085	660,086	349	159.505*	84.789*	30.944	12.828	37.9	90	81	37	8	137
***T. selloi***^**#**^	Frazão 235	10,758,439	407,552	443	158.543*	84.195*	30.791	12.766	38	90	81	37	8	137
*T. tetragonolobum*	Frazão 419	14,350,498	189,678	85	158.851	85.447	30.450	12.504	38	90	81	37	8	137
*T. tetramerum*	Pace 31	5,460,117	489,472	580	159.507	85.204	30.749	12.805	38	90	81	37	8	137
*T. truncatum*	Frazão 340	14,079,736	612,725	216	158.631	85.072	30.423	12.713	38	90	81	37	8	137
*T. xanthophyllum*	Frazão 333	14,242,787	600,064	341	160.935	86.213	30.961	12.800	37.8	90	81	37	8	137

**Table 3:** Genes encoded by the *Tanaecium* plastomes and their type and function. Asterisks (*) after gene names indicate genes with one intron, and double asterisks (**) indicate genes with two introns. Number one after gene names indicate genes duplicated.

Gene function	Gene type	Gene
Self-replication	Ribossomal RNA genes	*rrn*4.5^1^, *rrn*5^1^, *rrn*16^1^, *rrn*23^1^
	Transfer RNA genes	*trn*A-UGC*^,1^, *trn*C-GCA, *trn*D-GUC, *trn*E-UUC, *trn*F- GAA, *trnf*M-CAU, *trn*G-UCC, *trn*G-UCC*, trnH-GUG, *trn*I-CAU^1^, *trn*I-GAU*^,1^, *trn*K-UUU*, *trn*L-CAA^1^, *trn*L- UAA*, *trn*L-UAC, *trn*M-CAU, *trn*N-GUU^1^, *trn*P-UGG, *trn*Q-UUG, *trn*R-ACG^1^, *trn*R-UCU, *trn*S-GCU, *trn*S-GGA, *trn*S-UGA, *trn*T-GGU, *trn*T-UGU, *trn*V-GAC^1^, *trn*V-UAC*, *trn*W-CCA, *trn*Y-GUA
	Small ribosomal subunit	*rps*2, *rps*3, *rps*4, *rps*7^1^, *rps*8, *rps*11, *rps*12**^1^, *rps*14, *rps*15^1^, *rps*16*, *rps*18, *rps*19
	Large ribosomal subunit	*rpl*2*^,1^, *rpl*14, *rpl*16*, *rpl*20, *rpl*22b, *rpl*23^1^, *rpl*32, *rpl*33, *rpl*36
	RNA polymerase subunits	*rpo*A, *rpo*B, *rpo*C1*, *rpo*C2
Photosynthesis	Photosystem I	*psa*A, *psa*B, *psa*C, *psa*I, *psa*J
	Assembly/stability of photosystem I	*ycf*3**, *ycf*4
	Photosystem I	*psb*A, *psb*B, *psb*C, *psb*D, *psb*E, *psb*F, *psb*H, *psb*I, *psb*J, *psb*K, *psb*L, *psb*M, *psb*N, *psb*T, *psb*Z
	NADH dehydrogenase	*ndh*A*, *ndh*B*^,1^, *ndh*C, *ndh*D, *ndh*E, *ndh*F, *ndh*G, *ndh*H, *ndh*I, *ndh*J, *ndh*K
	Cytochrome b/f complex	*pet*A, *pet*B*, *pet*D*, *pet*G, *pet*L, *pet*N
	ATP synthase	*atp*A, *atp*B, *atp*E, *atp*F*, *atp*H, *atp*I
	Rubisco	*rbc*L
Other genes	Translational initiator factor	*inf*A
	Maturase	*mat*K
	Protease	*clp*P**
	Envelope membrane protein	*cem*A
	Subunit of Acetil-CoA-carboxylase	*acc*D
	c-type cytochrome synthesis	*ccs*A
Pseudogenes in some species		*ψrps*15, *ψycf*68
Unknown function	Hypothetical chloroplast reading frames	*ycf*1^1^, *ycf*15, *ycf*2, *ycf*68^1^

### Nucleotide diversity analyses

**Figure 3:**
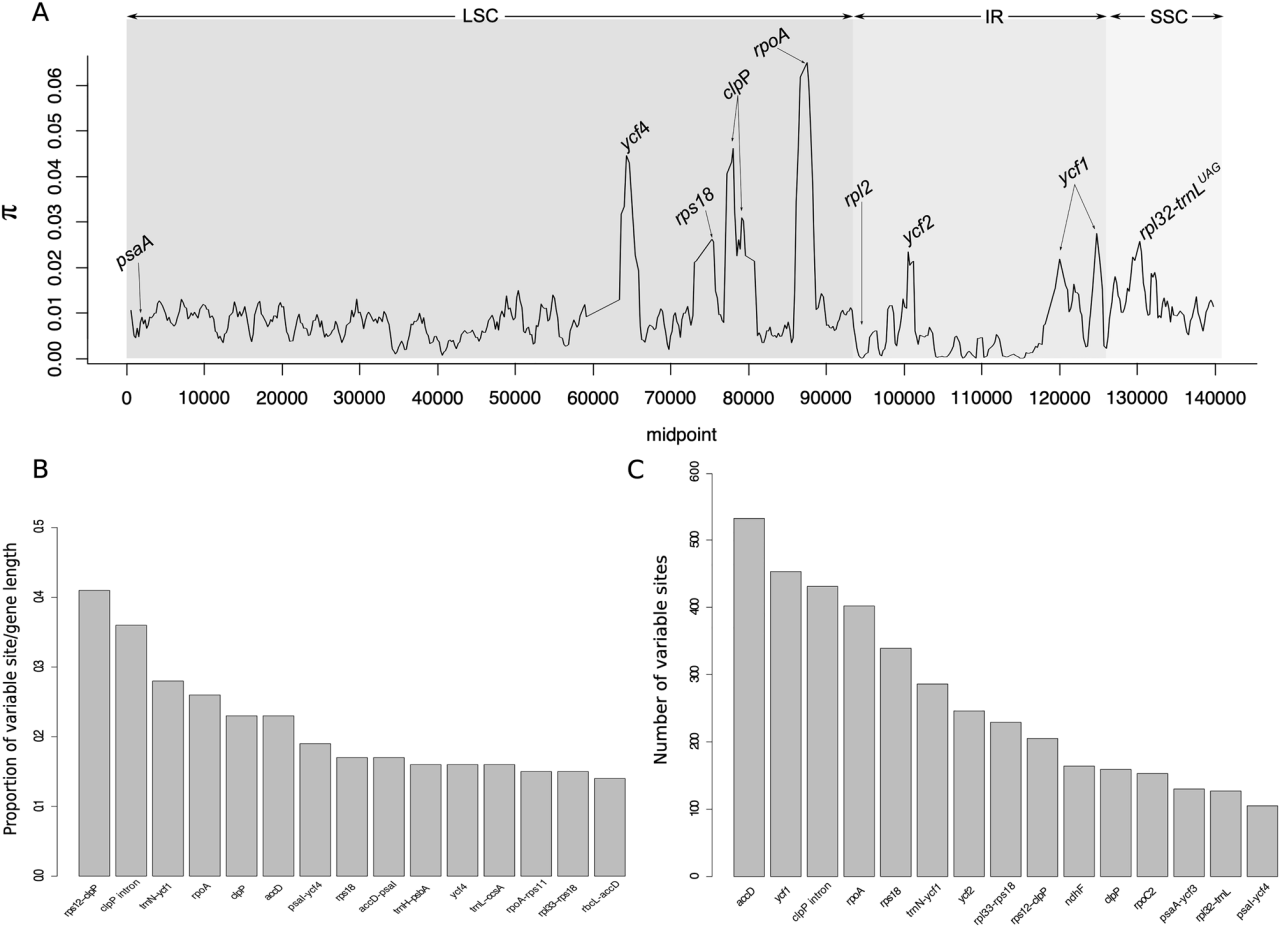
(**A**) Sliding window analysis of the complete plastomes of 15 *Tanaecium* species (window length: 800 bp, step size: 200 bp). X-axis, the position of the midpoint of each window. Y-axis, nucleotide diversity (π) of each window. (**B**,**C**) Fifteen most variable genes within the assembled *Tanaecium* plastomes. (**B**) Percentage of variable sites according to gene length. (**C**) Number of variable sites per gene.

The analysis performed using the DnaSP to calculate the nucleotide variability (π) values within 800 bp across plastomes showed that there is intrageneric variability in *Tanaecium* (Fig. 3A). The π values range from 0 to 0.06, with a mean value of 0.009. The most variable region, the only one containing π > 0.05, was the *rpoA* gene. Seven regions showed π values between 0.03 and 0.049 (i.e., *clp*P, *psa*I-*ycf*4, *pet*D-*rpo*A, *rps*11, *rps*12-*clp*P, *ycf*4, and *rpo*A), while twelve regions showed π > 0.02 (i.e., *ycf*2, *ycf*1, *rpl*33, *clp*P*-psb*B, *rpl*33*-rps*18, *rpl*32*-trn*L, *rpl*32, *clp*P, *ycf*4, *rpl*20*-rps*12, *rps*11, and *rps*18) (Fig. 3A). The non-coding regions are more variable (7.65% of the intergenic regions (IGS) and 6.05% of the introns) than the coding regions (5.75%; Supplementary Table S1). Among all plastome regions, the 15 regions with the highest percentage of variable sites are: *rps*12-*clp*P, *clp*P intron, *trn*N-*ycf*1, *rpo*A, *clp*P, *acc*D, *psa*I-*ycf*4, *rps*18, *acc*D-*psa*I, *trn*H-*psb*A, *ycf*4, *trn*L-*ccs*A, *rpo*A-*rps*11, *rpl*33-*rps*18, and *rbc*L-*acc*D (Fig. 3B; Supplementary Table S1). The 15 most variables regions in absolute numbers are: *acc*D, *ycf*1, *clp*P intron, *rpo*A, *rps*18, *trn*N-*ycf*1, *ycf*2, *rpl*33-*rps*18, *rps*12-*clp*P, *ndh*F, *clp*P, *rpo*C2, *psa*A-*ycf*3, *rpl*32-*trn*L, and *psa*I-*ycf*4 (Fig. 3C; Supplementary Table S1).

### Repeat analyses

**Figure 4:**
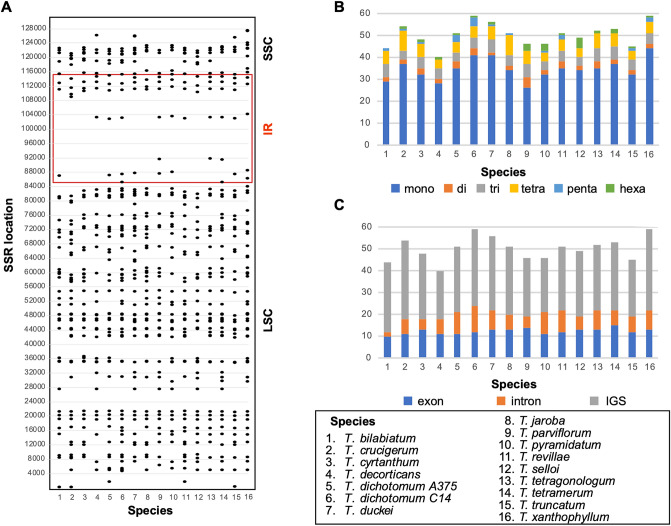
Distribution of SSRs in the *Tanaecium* plastomes. (**A**) Distribution of SSRs (IRa omitted). (**B**) Number of SSRs by type. (**C**) Distribution of SSR by coding and non-coding regions.

The total number of SSRs (i.e., tandem repeats of short motifs of DNA with lengths varying from 1 to 6 bp) in *Tanaecium* range from 44 to 59 SSRs, distributed along the three regions (Fig. 4A–C; Supplementary Table S2). Most SSRs found are A or T mononucleotide repeats, accounting for 54–73% of the total repeats. Out of the total number of SSRs detected, 26–44 (56.5–74.6%) are mono-repeats, 1–5 (1.8–10.9%) are di-repeats, 4–6 (7.4–13%) are tri-repeats, 4–9 (8.2–17.6%) are tetra-repeats, 0–4 (0–6.8%) are penta-repeats, while 0–5 (0–10.2%) are hexa-repeats (Fig. 4B; Supplementary Table S2). In addition, most of the SSRs in *Tanaecium* are located in the LSC region (71–82.4%). The IR regions include between 1.9 and 15.2%, while the SSC region includes between 4.3 and 27% of the SSRs (Fig. 4A; Supplementary Table S2). The coding regions contain 20.3–30.4% of the SSRs, while the introns contain 4.5–21.7%, and the intergenic spacers contain 54.3–72.7% of the SSRs (Fig. 4C; Supplementary Table S2).

**Figure 5:**
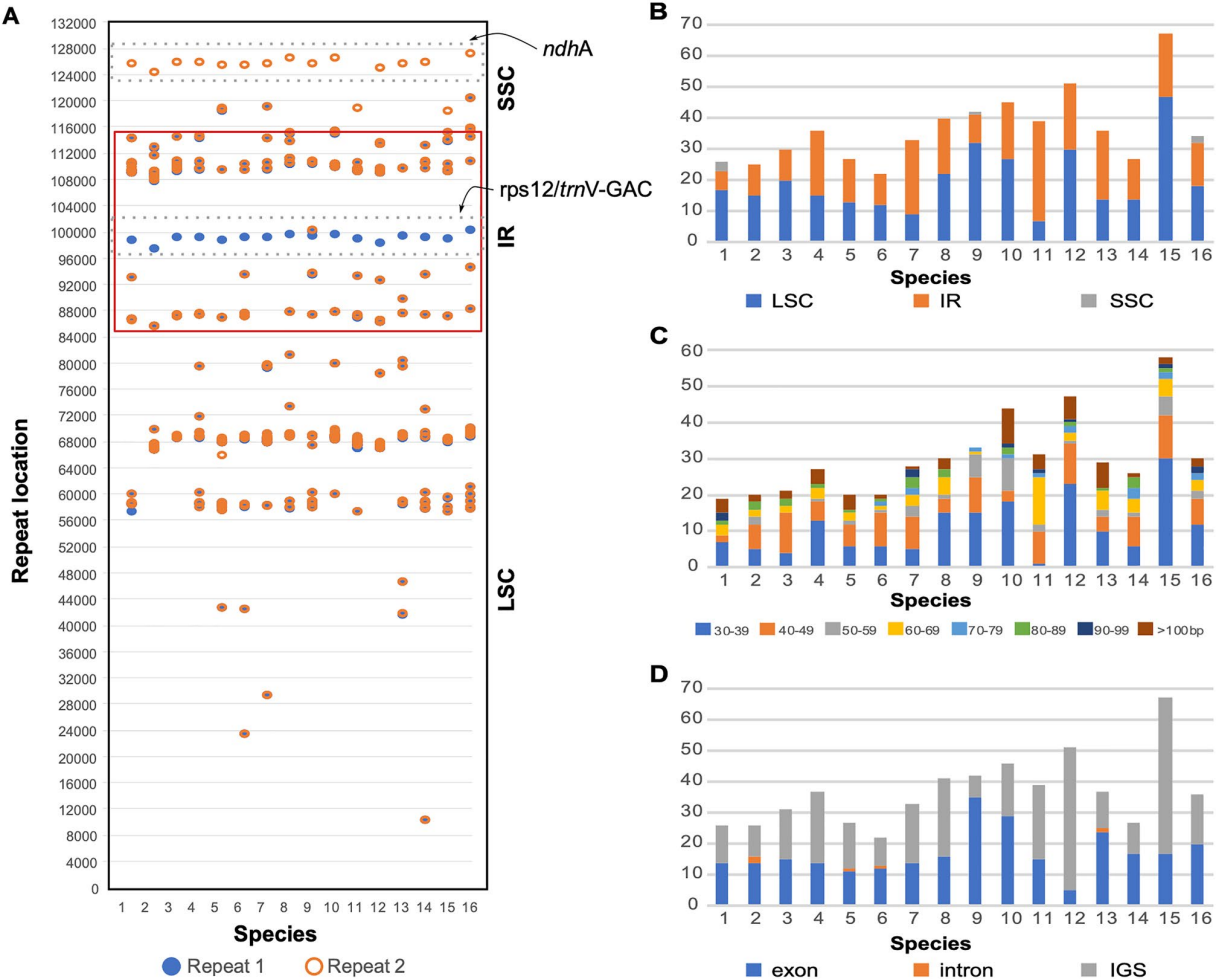
Distribution of tandem repeats, 30 bp or longer in the *Tanaecium* plastomes. (**A**) Distribution of the repeats (IRa omitted). (**B**) Distribution and size of the repeats along the unique regions of the plastome: Large Single Copy (LSC), Small Single Copy (SSC), and Inverted Repeat (IR). (**C**) Distribution of the repeats by size. (**D**) Distribution of the repeats by size and coding and non-coding regions.

We identified tandem repeat sequences longer than 30 bp throughout the *Tanaecium* plastomes (Fig. 5A; Supplementary Table S3). Most of these tandem repeats are found in the LSC regions, followed by the IR, with only a few tandem repeats found in the SSC (Fig. 5B; Supplementary Table S3). The most frequent repeats were 30–39 bp in length (Fig. 5C; Supplementary Table S3). Most of the tandem repeats are located in the IGS, followed by the CDS, while few repeats were found in introns (Fig. 5D; Supplementary Table S3). The plastomes of *Tanaecium* contain 20–67 forward repeats, up to two reverse repeats, and single palindromic repeats, leading to a total of 22–67 repeats (Supplementary Table S3). The longest repeats vary between 79 bp in *T. parviflorum* and 418 bp in *T. pyramidatum* (Supplementary Table S3). The longest repeats are located in eight regions: *acc*D, *rpo*A, *ycf*1, and *rps*18 genes, or the *rpl*23/*trn*I*-*CAU, *rpl*33/*rps*18, *psa*A/*ycf*3, and *trn*N-GUU/*ycf*1 intergenic regions (Supplementary Table S3). A shared repeat with 41 bp showed the first repeat in the intergenic region *rps*2/*trn*V-GAC, the second in the *ndh*A intron for all *Tanaecium* species, and four additional Bignonieae plastomes included in this study (Fig. 5A; Supplementary Table S4).

### Selection signature on plastomes

**Figure 6:**
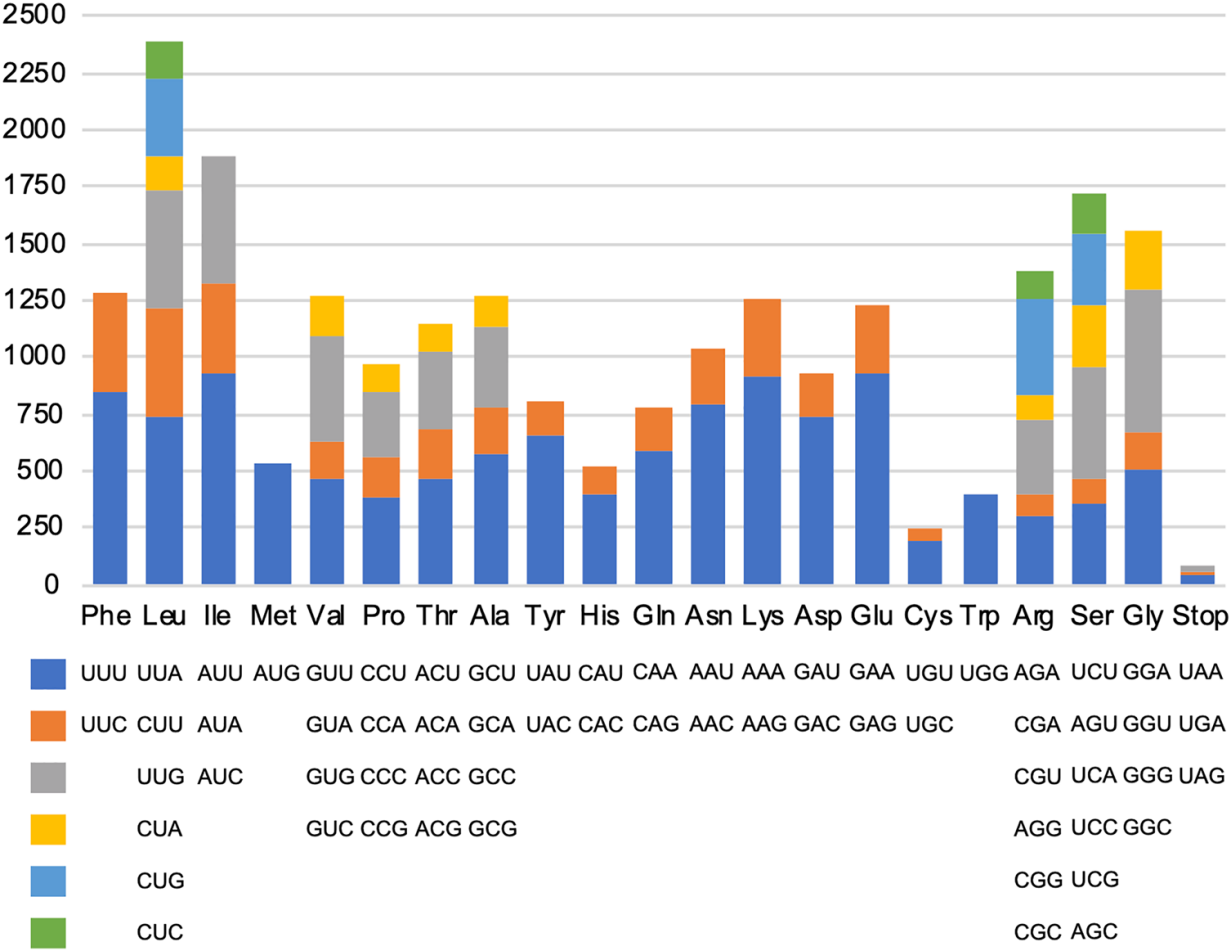
Codon content of amino acids encoding proteins in the chloroplast genomes of *Tanaecium*. All frequencies are averages over all taxa.

The 81 protein-coding genes of the *Tanaecium* plastomes encoded 22,686 codons averaged over all taxa (Supplementary Table S5). The most abundant codons encoded leucine (10.5%), followed by isoleucine (8.3%); whereas the least abundant codons encoded cysteine (1.07%), followed by the stop codons (0.35%) (Fig. 6). Thirty-two codons showed codon usage bias (RSCU < 1), of which only three are not G- and C-ending. Thirty codons were used more frequently than expected at equilibrium (RSCU > 1), with one not representing an A/U-end codon. Codon bias was not detected (RSCU = 1) in the frequency of use for the start codon AUG (methionine) and UGG (tryptophan) (Supplementary Table S5). None of the 81 genes were found to be under positive selection in *Tanaecium* using HyPhy^30^ in MEGA 7^31^. However, signals of positive selection were detected using the codon models BUSTED^32^ and FUBAR^33^ in eight coding regions: *acc*D (29 sites), *clp*P (15 sites), *rpo*A (39 sites), *rps*18 (15 sites), *rps*7 (2 sites), *ycf*1 (37 sites), *ycf*2 (71 sites), and *ycf*4 (9 sites) (Supplementary Table S6).

### Phylogenetic relationships within *Tanaecium*

**Figure 7:**
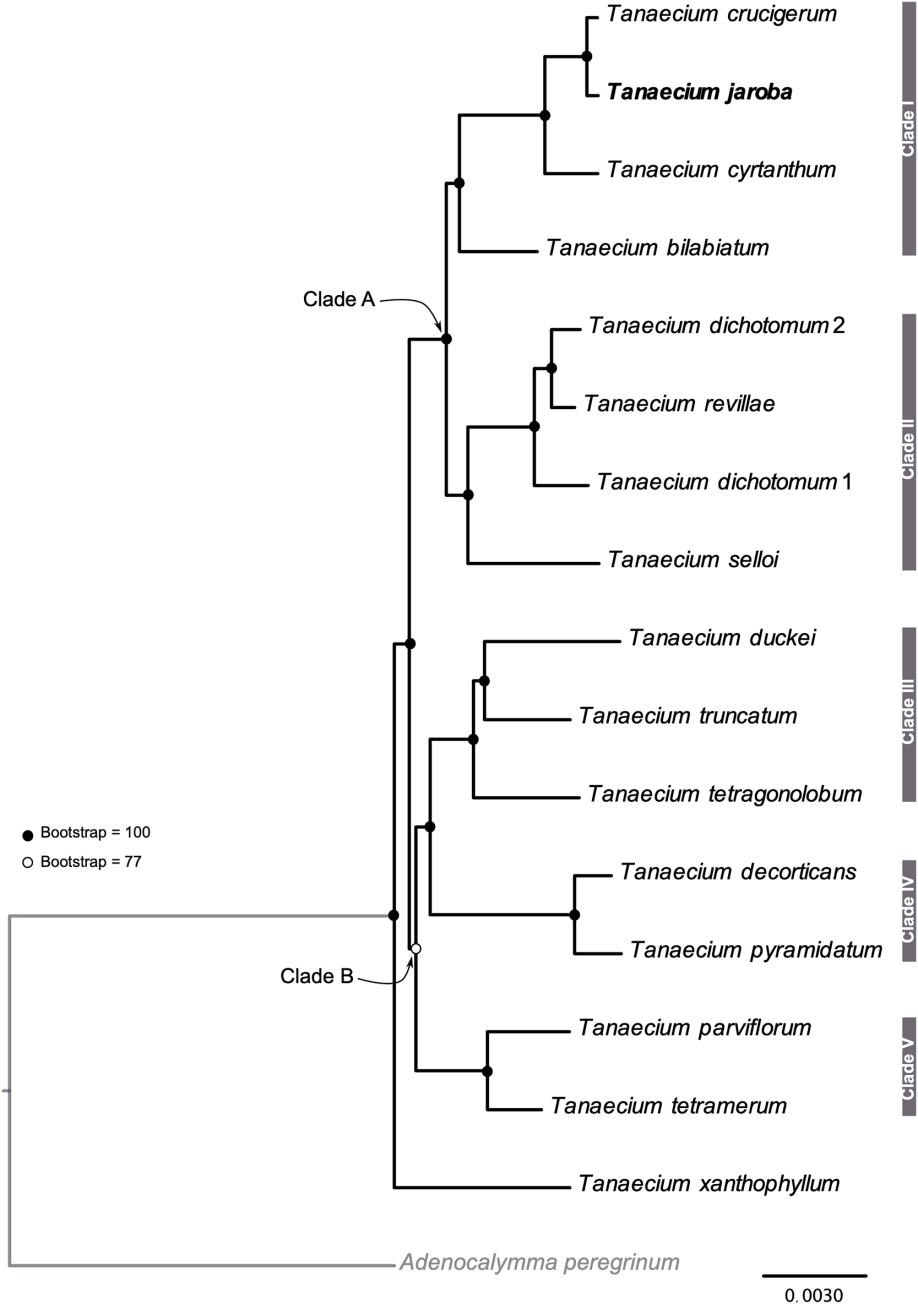
Maximum likelihood phylogeny inferred using IQ-TREE 1.5.5. The species highlighted in bold is the species type of the genus, *Tanaecium jaroba*.

The phylogeny of *Tanaecium* plus one outgroup was inferred using all 16 plastomes, removing one of the IRs and the poorly aligned regions. The final alignment included a total of 121,710 bp (86% of the original 140,117 positions), where 7,051 bp were variable and 2168 bp were parsimony informative. The best-fit model of substitution was the GTR + F + I + G4. The phylogeny recovered a monophyletic *Tanaecium*, with maximum support value (bootstrap support (BS) = 100; Fig. 7). Most nodes showed maximum support, with low to moderate values observed for only one node (BS = 77; Fig. 7). *Tanaecium xanthophyllum* emerged as sister-group to the remaining species, all of which are divided in two main clades: Clades A and B. Clade A comprises Clade I (i.e., *T. bilabiatum*, *T. crucigerum*, *T. jaroba*, and *T. cyrtanthum*) and Clade II (i.e., *T. selloi*, *T. dichotomum* 1, *T. revillae*, and *T. dichotomum* 2). In turn, Clade B is composed of Clade III (i.e., *T. tetragonolobum*, *T. truncatum*, and *T. duckei*), sister to Clade IV (i.e., *T. pyramidatum* and *T. decorticans*), both of which are sister to Clade V (i.e., *T. tetramerum* and *T. parviflorum*) (Fig. 7).

## Discussion

In this study, we sequenced and assembled for the first time 16 plastomes representing 15 of the 21 *Tanaecium* species currently recognized^21^. These plastomes were compared with previously published Bignoniaceae plastomes, providing novel insights into chloroplast evolution in the family. The newly assembled plastomes were used as a basis to reconstruct the most comprehensive phylogeny of *Tanaecium* to date. The phylogenetic placement of *Tanaecium jaroba*, the type species of the genus, was inferred for the first time, corroborating the current generic classification^21^.

The quadripartite plastome structure found in *Tanaecium* is the most common among angiosperms^3,7,8,34^. Some exceptions for this structure have been reported in the papilionoid legumes^35^, saguaro cactus^36^, and Geraniaceae^37^. Although plastome structural changes have been reported for angiosperms^6,8^, including tribe Bignonieae^20^, no rearrangement had ever been documented for *Tanaecium*. The two different patterns of boundaries between the four main regions found in *Tanaecium* plastomes are similar to that found in *Adenocalymma peregrinum*^20^ (Fig. 2). Contractions and expansions of IRs were detected multiple times during land plant evolution^38^, including other Bignoniaceae^16,19,20,28^. Within this plant family, the plastomes of *Bignonia magnifica* bear exceptionally large IR regions, representing the largest plastome among all Lamiids known to date^28^.

The obtained *Tanaecium* plastomes show a pattern of size range variation that matches that of the LSC expansions/contractions (Table 2). This is a typical pattern among seed plants, although the number of genes and intergenic region length is more commonly used to explain plastome size variation^10^. In other Bignonieae, the LSC size variation is relatively common^16,19,20^, and the variation in gene number seems less frequent for the group^16,19,20^.

When the Bignonieae IQR and median size variation ratio are compared with those expected for other angiosperms^9^, *Tanaecium*, *Adenocalymma*, and *Anemopaegma* show less than 1% variation at the genus level as reported for other groups^9^ (Supplementary Table S7), while *Amphilophium* shows variation greater than 4%^9^ (Supplementary Table S7). Even though the high variation found in *Amphilophium* was previously attributed to polyphyly^9^, this interpretation was based on an outdated classification system. *Amphilophium* monophyly has been shown repeatedly^39,40^. In this context, we attribute the high IQR and median size variation ratio found in *Amphilophium* to the gene number and LSC length variation^10,16^.

The total number of genes found in *Tanaecium* plastomes is similar to those found in other Bignoniaceae^16,20^. While *ycf*15 and *ycf*68 genes are lacking in some Bignoniaceae genera^16,19,20^, those genes were found in *Tanaecium*, *Callichlamys latifolia* (Rich.) K.Schum.^25^, and *Crescentia cujete* L.^29^. Partial *ycf*15 genes were also recorded in the Convolvulaceae^41^. The complete or partial loss of genes is common in land plants^6,9,10^, including the Bignoniaceae^20^.

The most variable locus in *Tanaecium* is *rpo*A, which contains hypervariable sites with π > 0.05. This gene is frequently listed among the most variable regions in other plant clades^42^ and has been shown to represent one of the most hypervariable genes for *Amphilophium* (Bignoniaceae)^16^. In turn, the *acc*D gene is the most variable in terms of absolute numbers in *Tanaecium* (Fig. 3), and the second most variable in *Amphilophium*, followed by the *ycf*1 gene^16^. The *acc*D gene is highly variable in other Bignoniaceae species and angiosperm clades such as *Artemisia* (Asteraceae)^43^ and *Lamprocapnos* (Papaveraceae)^8^. The *rps*18 gene is among the most variable in absolute numbers in *Tanaecium*, Stemonaceae^44^, Bromeliaceae^45^, and Campanulaceae^17^. Interestingly, the *rps*18 gene shows low evolutionary rates in *Anemopaegma* (Bignoniaceae)^19^, indicating that chloroplast genes can hold different levels of variation in distinct lineages and at different taxonomic levels. This aspect complicates the selection of candidate barcode genes for the angiosperms as a whole, emphasizing the importance of studies aiming to characterize plastomes of entire clades.

Single Sequence Repeats (SSRs) are commonly detected in plastomes, often showing interspecific polymorphism, and high variation at lower taxonomic levels, representing useful tools for population-level studies^46^. The SSRs identified in *Tanaecium* vary in location, type, and number. Most SSRs are located in the LSC region, with the mononucleotide A/T repeats representing the most abundant type (Fig. 4). The higher frequency of mononucleotides is a common trend among land plants^47^. Most of the long repeats of *Tanaecium* are located in the LSC, followed by IR regions, with only a few located in the SSC. This pattern differs from that found in other Bignoniaceae species, where most of the larger than 30 bp repeats are located in the IR, with only a few cases showing a pattern that is similar to that found here^16,48^. The chloroplast SSRs detected in *Tanaecium* will likely be helpful for future population genetics and microevolutionary studies, as well as for community-level studies of potential barcode designs, given the presence of shared repeats.

Plastomes have a synonymous codon usage bias in the protein-coding genes, which affects gene expression and plays an essential role in the evolution of these genomes^49^. Our results showed that amino acids that have A- and U-ending codons are more common in *Tanaecium*, consistent with codon usage bias in most of the angiosperm plastomes, including Bignoniaceae representatives^48,50^. In plants, the main evolutionary driving force acting on codon use are natural selection and mutation pressure^51–53^. Thus, the patterns observed in *Tanaecium* bring important information not only about the nature of plastome mutations, but also about putative environmental impact. More expressed genes might display higher codon bias^54^, which can be seen in plastomes due to the photosynthetic machinery associated with the chloroplast function. Our results also showed a preference for using the amino acid leucine, which has a high RSCU (Fig. 6; Supplementary Table S5), suggesting a potential impact of selection pressure on codon usage^51,54^.

Adaptive evolution or positive selection is generally estimated using the synonymous/non-synonymous substitutions ratio^55^. Even though our analyses using a maximum likelihood approach in HyPhy have failed to detect any signal of positive selection, evidence for positive selection was recovered through the analyses conducted with BUSTED and FUBAR. This result likely reflects the fact that a relatively high fraction of sites (5–10%) needs to be under positive selection for accurate detection in BUSTED^32^, while FUBAR assumes that the selection pressure for each site is constant throughout the phylogeny^33^. Thus, it is likely that the genes really have evidence for selection. For the eight genes under positive selection in *Tanaecium*, seven of them were also shown to be under positive selection in *Amphilophium* (except *ycf*4)^16^, while three were shown to be under positive selection in *Handroanthus impetiginosus* (Mart. ex DC.) Mattos (i.e., *rps*7, *ycf*1, and *ycf*4)^48^. The genes found under selection are associated with different plant cell functions. They are associated explicitly with ribosome biogenesis and protein synthesis^56^, RNA polymerase biogenesis^57^, assembly and stability of the photosystem I^58^, environmental stress and plant growth^59^, among other important components of cell function and survival^60,61^.

The ML phylogeny reconstructed here sampled 15 out of the 21 currently accepted species of *Tanaecium*, representing the most comprehensive phylogeny of the genus to date, regarding the number of characters and taxa. A previous topology was inferred to investigate the relationship of a recently described *Tanaecium* species, sampling 11 species of the genus and using only the nuclear marker *pep*C and the chloroplast gene *ndh*F^21^. The sampling used here is different, making comparisons among the resulting topologies difficult. In addition, some relationships were not clearly solved in the previously published tree reconstructed with two markers, with several nodes showing low/moderate support^21^. Yet, the placement of the newly described species in that study was similar to the one inferred here (i.e., *T. decorticans* + *T. pyramidatum*). Moreover, the phylogeny inferred here is the first to include the type species of the genus (i.e., *T. jaroba*), confirming the monophyly of the genus hypothesized earlier^12^. Our results indicate that the variation found among plastomes is sufficient to reconstruct robust phylogenetic relationships of the 16 *Tanaecium* taxa sampled here with good support. Additional studies will be released soon, further investigating the phylogenetic relationships among *Tanaecium* species, their morphological evolution, and biogeographical history.

## Materials and methods

### Taxon sampling, DNA extraction, genomic sequencing, plastome assembly, and annotation

We sequenced, assembled, and annotated the plastomes of 15 out of 21 species of *Tanaecium* currently recognized^21^, namely: *Tanaecium bilabiatum* (Sprague) L.G.Lohmann, *Tanaecium crucigerum* Seem., *Tanaecium cyrtanthum* (Mart. ex DC.) Bureau & K.Schum., *Tanaecium decorticans* Frazão & L.G.Lohmann, *Tanaecium dichotomum* (Jacq.) Kaehler & L.G.Lohmann, *Tanaecium duckei* A.Samp., *Tanaecium jaroba* Sw., *Tanaecium parviflorum* (Mart. ex DC.) Kaehler & L.G.Lohmann, *Tanaecium pyramidatum* (Rich.) L.G.Lohmann, *Tanaecium revillae* (A.H.Gentry) L.G.Lohmann, *Tanaecium selloi* (Spreng.) L.G.Lohmann, *Tanaecium tetragonolobum* (Jacq.) L.G.Lohmann, *Tanaecium tetramerum* (A.H.Gentry) Zuntini & L.G.Lohmann, *Tanaecium truncatum* (A.Samp.) L.G.Lohmann, and *Tanaecium xanthophyllum* (DC.) L.G.Lohmann. We sampled two individuals of *T. dichotomum*, representing different morphotypes of this species (i.e., *Tanaecium dichotomum* 1 and *Tanaecium dichotomum* 2). All sampled taxa, vouchers, and respective GenBank accession numbers are summarized in Table 1.

Leaf tissue was pulverized with Tissuelyzer^®^ (Qiagen, Duesseldorf, Germany) for 5 min at 50 Hz and DNA was subsequently extracted following the CTAB protocol^62^. The protocol was adapted by adding 2-Mercaptoethanol and polyvinylpyrrolidone (PVP). DNA was quantified using the Qubit^®^ Fluorometer (Thermo Fisher Scientific, Waltham, MA, USA). A total of 5 μg of DNA was fragmented using a Covaris S-series sonicator, generating DNA fragments of approximately 300 bp. Libraries for Illumina platform sequencing were prepared following Nazareno et al.^25^ Sequencing was conducted in an Illumina HiSeq 2500 Genome Analyzer (Illumina, San Diego, California, USA) as paired-read, with 22 samples per lane, at USP-Esalq (Piracicaba, Brazil).

Plastomes were assembled using the Fast-Plast pipeline (McKain and Wilson, unpubl.; https://github.com/mrmckain/Fast-Plast). This pipeline uses Trimmomatic 0.35^63^ to remove the adaptors and low-quality sequences. The trimmed reads were mapped against a database that included the published plastomes of *Adenocalymma peregrinum* (MG008314.1), *Olea europaea* L. (NC_013707.2), *Sesamum indicum* L. (NC_016433.2), *Salvia miltiorhiza* Bunge (NC_020431.1), and *C. latifolia* (KR534325) using Bowtie 2.1.0^64^. Mapped reads were assembled into contigs using SPAdes 3.1.0^65^. Resulting contigs were assembled with the software afin (https://bitbucket.org/afinit/afin), using the parameters -l 50, -f 0.1, -d 100, -× 100, and -i 2. For species for which it was harder to obtain comprehensive contigs, we tested values between 10 and 20 for minimum contig (-p) parameter overlap. The final assembly from Fast-Plast or afin was checked, and edited with Geneious 9.0.2^66^. The plastome assembly was verified through a coverage analysis conducted in Jellyfish 2.1.3^67^ using a 25-bp sliding window of coverage across the plastome of each species. Only sites with a depth higher than two were kept.

Plastome annotation was initially conducted in Geneious 9.0.2^66^ using the *Adenocalymma peregrinum* plastome as a reference^20^. The annotated loci were verified using BLAST^68,69^, with correct start and stop codons of the Open Reading Frames (ORFs) checked manually in Geneious 9.0.2^66^. The boundaries between the LSC, IRs, and SSC regions were verified using the online IRscope^70^ and confirmed manually in Geneious 9.0.2^66^. The graphical representation of the annotated *Tanaecium* plastomes was created using OGDRAW^71^.

### Plastome comparative analyses

We performed comparative analyses using the 16 *Tanaecium* plastomes sequenced (Table 1). We removed one of the IR regions from all plastomes to avoid data duplication, except for the analyses to determine synteny and identify possible rearrangements which were conducted for the complete plastomes using Mauve 2.4.0^72^. These analyses utilized mauveAligner as alignment algorithm, MUSCLE 3.6^73^ as the internal aligner, with full alignment and minimum locally collinear block (LCB) score automatically calculated. Genomes were not assumed to be collinear. We used the online IRscope^70^ to compare *Tanaecium* plastome borders between the four main regions (i.e., LSC, IRs, and SSC) within the genus and with other five previously published Bignonieae plastomes: *Adenocalymma peregrinum* (MG008314.1), *Amphilophium steyermarkii* (MK163626), *Anemopaegma arvense* (MF460829), *Callichlamys latifolia* (KR534325.1), and *Crescentia cujete* (KT182634) (Table 1). To compare the length variation of *Tanaecium* plastomes and other Bignonieae genera with previously published plastomes, we used the box-plot approach proposed by Turudić et al.^9^.

*Tanaecium* plastomes were aligned in MAFFT 7 online version^74^ where analyses of intrageneric variability were conducted. The poorly aligned regions were removed using Gblocks 0.91b^75^, assuming the least stringent settings. We calculated nucleotide variability values (π) within the assembled *Tanaecium* plastomes using DnaSP 6.10^76^ through a sliding window analysis with a 200 bp step size and 800 bp window length. We used R^77^ to plot the DnaSP results. We extracted annotated coding and non-coding regions using Geneious 9.0.2^66^ to evaluate the number of variable sites (V) using the software MEGA 7^31^. The protein-coding regions were previously re-aligned individually with the translation alignment tool in Geneious 9.0.2^66^ using the ClustalW plugin^78^.

### Analyses of the repeated regions

To identify and locate microsatellites or Simple Sequence Repeats (SSRs) in *Tanaecium* plastomes, we used MISA^79^ with the following parameters: motif length of SSR between one and six nucleotides, a minimum repetition number set as 10 units for mono-, five for di-, and four for trinucleotide SSRs, and three units for each tetra-, penta-, and hexanucleotide SSRs. We used REPuter^80^ to identify tandem repetitions, allowing forward, palindrome, and reverse repeated elements with a minimum repeat size ≥ 30 bp and Hamming distance of 0.

### Plastome codon usage and signature of molecular selection

To investigate the codon usage and the role of selection on *Tanaecium* plastomes, we extracted 81 protein-coding genes from the 16 genomes aligned and annotated. Each coding region was re-aligned separately in Geneious^66^, using the translation alignment tool ClustalW plugin. Codon usage bias occurs when some codons are used more often than other synonymous codons during gene translation between different taxa^81^. We assessed the relative synonymous codon usage (RSCU) from the 81 protein-coding genes using MEGA 7^31^, with default parameters.

In addition, we investigated synonymous (Ks) and non-synonymous (Ka) substitutions and their ratio (Ka/Ks) in the 81 coding regions using the package HyPhy^30^ in MEGA 7^31^. We also used other codon models to further analyze the selective pressure on the protein-coding genes using HyPhy^30^ in the Datamonkey server^82^: i.e., BUSTED (branch-site unrestricted statistical test for episodic diversification; Murrell et al.^32^) was used to investigate diversifying selection on the selected genes, while FUBAR (fast unconstrained Bayesian AppRoximation; Murrell et al.^33^) was used to identify episodic/diversifying selection on codon sites with posterior probability of > 0.9.

### Phylogeny reconstruction

The 16 plastomes of the 15 *Tanaecium* species assembled here were aligned using the *Adenocalymma peregrinum* (MG008314) plastome as an outgroup and the online version of MAFFT 7^74^. The Ira regions were excluded from the alignment to avoid data duplication. We used Gblocks to remove poorly aligned regions with the least stringent settings^75^. The number of variable and parsimony informative sites for the resulting alignment was calculated in MEGA 7^31^. The final alignment was used to perform maximum likelihood (ML) analyses in IQ-TREE 1.5.5^83^, including model selection and 1000 bootstrap (BS) replicates in a single run^84^.

## Supplementary Information

Supplementary Figure S1.

Supplementary Tables.

## Data Availability

The assembled plastomes of *Tanaecium* are available in GenBank (NCBI) with the accession numbers OL782596, OP169019–OP169021, and OP218850–OP218861.
