# Supplementary figures and images for: Comparative chloroplast genomics and insights into the molecular evolution of Tanaecium (Bignonieae, Bignoniaceae)
Source: Sci Rep. 2023 Aug 1;13:12469. doi: 10.1038/s41598-023-39403-z (PMC10394017; doi:10.1038/s41598-023-39403-z)

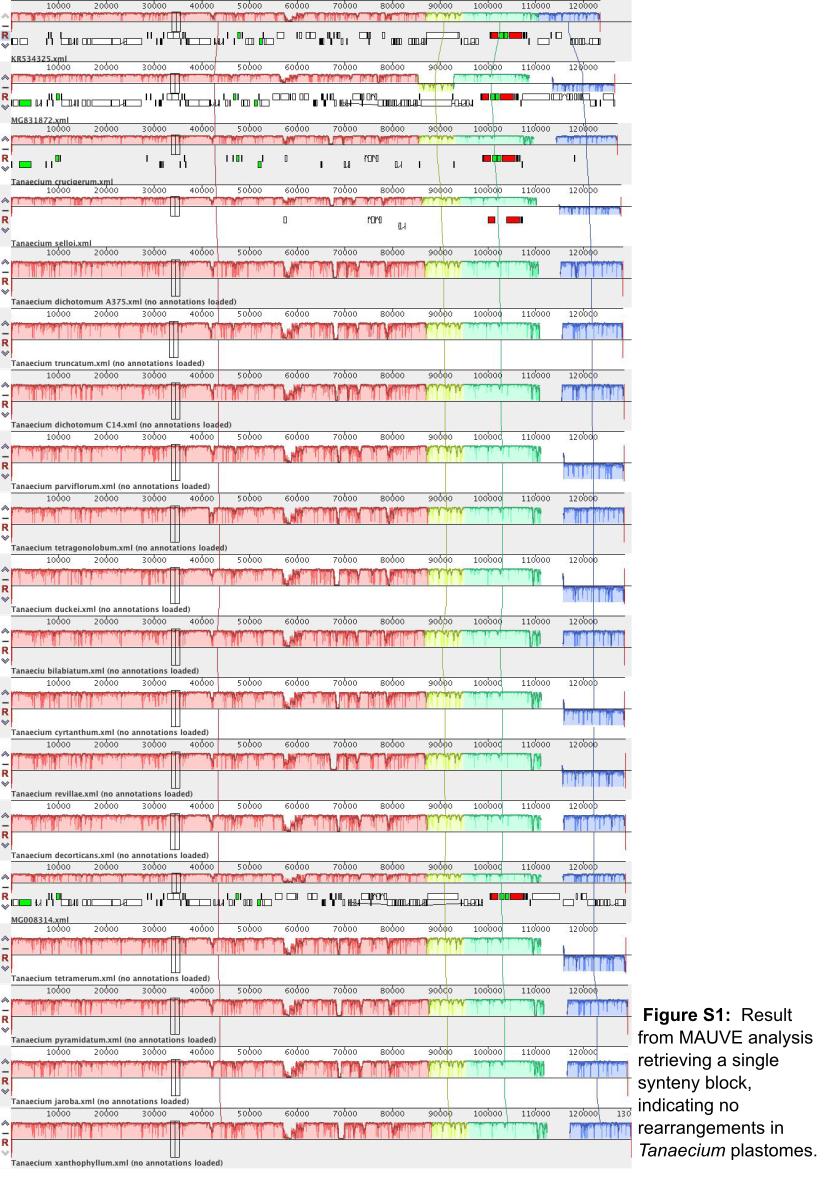

Supplement: Supplementary file 1 — Supplementary Figure S1. [file 41598_2023_39403_MOESM1_ESM.png]
